# Functional impairment among people diagnosed with depression in primary healthcare in rural Ethiopia: a comparative cross-sectional study

**DOI:** 10.1186/s13033-019-0305-8

**Published:** 2019-07-17

**Authors:** Kassahun Habtamu, Girmay Medhin, Medhin Selamu, Kebede Tirfessa, Charlotte Hanlon, Abebaw Fekadu

**Affiliations:** 1 School of Psychology, College of Education and Behavioral Studies, Addis Ababa University, P.O.BOX: 1176, Addis Ababa, Ethiopia; 2 Aklilu Lemma Institute of Pathobiology, Addis Ababa University, Addis Ababa, Ethiopia; 3 Department of Psychiatry, School of Medicine, College of Health Sciences, Addis Ababa University, Addis Ababa, Ethiopia; 4 Health Services and Population Research Department, Institute of Psychiatry, Psychology and Neuroscience, Centre for Global Mental Health, Kings College London, London, UK; 5 Department of Psychological Medicine, Institute of Psychiatry, Psychology and Neuroscience, Centre for Affective Disorders, King’s College London, London, UK; 6 Global health & Infection, Brighton and Sussex Medical School, Brighton, UK

**Keywords:** Depression, Depressive symptoms, Functional impairment, Disability, Primary health care, Low- and middle-income countries

## Abstract

**Background:**

There have been few studies examining the functioning of clinically-diagnosed people with depression in primary healthcare (PHC) in low- and middle-income countries (LMICs). The aim of this study was to identify factors associated with functional impairment among people diagnosed with depression in PHC in Ethiopia as part of implementation of a task-shared model of mental healthcare.

**Methods:**

A comparative cross-sectional study was conducted. As part of the Programme for Improving Mental health carE (PRIME), PHC clinicians were trained to diagnose depression using an adapted version of the World Health Organization (WHO) mental health Gap Action Programme (mhGAP). A total of 2038 adult consecutive PHC attendees were screened for depressive symptoms using the 9-item Patient Health Questionnaire (PHQ-9). Those who scored five or above on the PHQ-9 (n = 131) were assessed by PHC workers. Of these, 92 were diagnosed to have depression (“PHC diagnosed cases”) and the remaining 39 people were PHQ positive but considered not to have depression (“non-diagnosed controls”). PHC diagnosed cases were also compared to a community representative sample of adult healthy controls (n = 197; “community controls”). The 12-item version of the WHO Disability Assessment Schedule (WHODAS-2.0) was used to assess functional impairment. Multivariable negative binomial regression models were fitted to examine the association of demographic, social, economic and clinical characteristics with functional impairment.

**Results:**

No significant difference in functional impairment was found between diagnosed cases and non-diagnosed controls. PHC diagnosed cases were found to have higher depressive symptom severity and suicidality, but lower social support compared to non-diagnosed controls (P < 0.05). In the multivariable model, greater functional impairment was associated with higher depressive symptoms (RR = 1.04; 95% CI 1.02, 1.05) and lower social support (RR = 0.96; 95% CI 0.95, 0.98). Diagnosed cases were found to have higher functional impairment compared to community controls (RR = 1.91; 95% CI 1.74, 2.09).

**Conclusion:**

In this study, PHC clinicians identified cases of depression with high symptom burden, suicidality and functional impairment. These findings support current initiatives to scale-up mental health services at the PHC level; and indicate that social support is an important target for intervention.

## Background

A diagnosis of depression requires the experience of depressed mood, loss of interest and enjoyment, and/or reduced energy leading to diminished activity for at least 2 weeks, as well as functional impairment or subjective distress [1]. Although the prevalence of depression varies between populations, every year an estimated 5.8% of the adult population develops a depressive episode and the lifetime risk for severe depression is estimated to be 12–16% [2]. Overall, depressive disorders are the leading cause of disability globally [3]; and major depressive disorder is the second leading cause of the global burden of disease [4]. Depression accounted for 4.5% of the worldwide total burden of disease in 2007 and is also responsible for the greatest proportion of burden attributable to non- fatal health outcomes, accounting for almost 12% of total years lived with disability worldwide [5].

In studies from both high income countries and low- and middle-income countries (LMICs), depression was found to be associated with significant functional impairment, decreased quality of life, increased use of health services, higher degree of morbidity, elevated risk for mortality [6, 7] and a compromised overall health status [8]. Evidence suggests that roughly 60% of people with depression reported substantial (severe or very severe) functional impairment [9]. Using data from the World Health Survey, Moussavi and colleagues [8] found that depression results in the greatest disability compared with other chronic diseases, including angina, arthritis, asthma, and diabetes. Depression also brings about societal burdens [10, 11], including disability in terms of lost wages, low productivity and impaired interpersonal relationships.

Epidemiological studies, mostly from high income countries, show that several factors are associated with functional impairment or disability among people with depression. These include, demographic factors (such as lack of marital life), and lower socio-economic status (lower education and poverty) [12]; factors related to interpersonal relationships (particularly lack of social support) [13]; clinical characteristics, including severity of depressive symptoms [14], longer duration of untreated depression [15] and cognitive dysfunction [16–19]. Cross-sectional studies conducted in Uganda and Rwanda found significant association between severity of symptoms of depression and difficulty performing relevant day to day activities which are crucial for survival [10, 11]. A study on outcome of major depression in rural Ethiopia showed that individual disability domain scores were significantly greater for those with persistent depression than for those who had completely recovered [20].

The prevalence of depression is high in the primary health care (PHC) setting [21, 22]. In a large study of PHC attendees in 14 countries, the prevalence of depression, on average, was 24% [23]. Similar levels are seen in studies from Africa [24]. However, the detection of depression in the PHC setting is low. A meta-analysis of 41 studies found that about half of the cases with depression are undetected by PHC clinicians [25]. The few studies in Africa indicate that the level of detection of depression in primary care is unacceptably low [24]. A recent study in rural Ethiopia found the detection rate of depression by primary care clinicians to be extremely low, with over 95% of patients presenting to primary care with potential depression not receiving a clinical diagnosis of depression [26]. The low detection of depression in primary care settings contributes to the high treatment gap in LMICs [27]. Integrated care (a task-sharing care approach provided at the PHC level by non-specialists) is the strategy proposed to address the treatment gap in LMICs [1]. However, evidence on the level of functional impairment and associated factors among diagnosed cases of depression at the primary healthcare (PHC) setting is lacking. Furthermore, little is known about the extent to which PHC clinicians consider functional impairment when making a diagnosis of depression.

This study was conducted as part of the PRogramme for Improving Mental health carE (PRIME), which aimed to generate evidence on the integration of mental healthcare in five LMICs [28]. The study aimed at identifying the factors associated with functional impairment among people who are diagnosed with depression by PHC clinicians who have been trained as part of a task-sharing model of care. The study also compared functional impairment of PHC diagnosed cases with non-diagnosed controls but found to have depressive symptoms as well as to community representative healthy controls. We hypothesized that lack of marital life, lower socio-economic status, lack of social support, experiencing a threatening life event, having a PHC diagnosis of depression and symptom severity would be associated with functional impairment.

## Methods

### Study design

A comparative cross-sectional study was conducted. A facility-based sample of individuals who were diagnosed as having depression was compared to (1) a sample who were not diagnosed but had depression symptoms, and (2) a community-based representative sample of healthy individuals.

### Study setting and context

The study was conducted in the Sodo district of the Gurage Zone, Southern Nations, Nationalities and Peoples’ Region (SNNPR). Sodo district is 100 km south of the capital city of Ethiopia, Addis Ababa. The district has a total population of 161,952 people (79,356 men and 82,596 women) [31]. Around 90% of the population lives in rural areas. Sodo district is the second largest in the SNNPR in terms of population size and the largest in land size [29]. Amharic is the official language in the district. Sodo Gurage is the largest ethnic group in the district (85.3%) and the majority of the population is Orthodox Christian (97%) [26]. The district has 8 health centers and 58 health posts [30]. Health centers are staffed by nurses, health officers, and midwives, who are trained at degree or diploma level. Each health center provides primary healthcare for 20,000–25,000 people. Health center clinicians deliver services such as diagnosis and treatment of communicable diseases (e.g. malaria, tuberculosis, and water-borne diseases), family planning, antenatal care, sanitation advice, malaria prevention, and give advice on the effects of harmful traditional practices. There is one general primary care hospital in the district, which had just opened at the time of the study.

At the time of this study, the nearest specialist mental health service for the district was found in Butajira town, 30 km south of Bui town, which is the capital of the Sodo district. However, with the support of the PRIME project, an integrated mental health care programme was started in Sodo district [29]. The PRIME mental health care plan included community, facility and organizational level interventions [31]. At the health centre level, PHC clinicians (health officers, nurses and mid-wives who are degree or diploma level graduates) were trained for 10 days using the World Health Organisation mental health Gap Action Programme (mhGAP) training materials (5 days) and on-the-job training (5 days) in the psychiatric out-patient clinic in Butajira hospital. PHC clinicians were supported with regular supervision from a psychiatric nurse. mhGAP focuses on equipping PHC clinicians to detect, assess and treat priority mental, neurological and substance use disorders, including depression. Sodo district was selected as a PRIME study area because it represents the geographical and cultural diversity of the country [31].

### Participants and recruitment

Experienced diploma holder lay data collectors, who were trained for 1 week, screened consecutive consenting adult attendees for depression at the eight health centers in Sodo district (n = 2038) during the study period February 03, 2015 to December 11, 2015. The inclusion criteria were age 18 years and above, resident in the district for 6 months or more and able to speak Amharic (the local official language) as Amharic was the language of interview. Those who scored above the validated cut-off for depression on the 9-item version of the Patient Health Questionnaire (PHQ-9; see below for measure details) were then assessed and diagnosed by general PHC clinicians. Those who were diagnosed with depression by PHC staff (“PHC diagnosed cases”) as well as those who were PHQ positive but not diagnosed by PHC staff (“non-diagnosed controls”) were included in the study.

To compare functional impairment between people who were diagnosed to have depression and individuals who had no depression, a community control group was used (“community controls”), which had been recruited for another study [32]. A total of 284 healthy controls were recruited, each member matched in terms of age (± 5 years), sex, village, household position (head vs. not head) and household size to a respondent from a household where a person with severe mental disorder (SMD) resided. Of these, those who had depressive symptoms (PHQ score ≥ 5) were excluded and the remainders were included in the study. The eligibility criteria included age 18 years or above, with no suspected or confirmed mental illnesses in the person or family member, PHQ score < 5 and providing consent to participate in the study. If more than one match was identified, one household was selected by lottery. If no respondent was identified from the first matched household after three home visits, or if they declined to participate, the next reserve was selected.

### Assessment of functional impairment and potential associated factors

We used the 12-item interviewer administered version of the World Health Organization Disability Assessment Schedule (WHODAS-2.0) to assess functional impairment. The WHODAS-2.0 is a cross-cultural, standardized and non-health condition specific measure of disability and functional impairment developed by the WHO [33]. It measures the activity limitations and participation restrictions of a person over the last 30 days [34]. The WHODAS-2.0 has been translated into several languages and adapted in many cultural contexts [35, 36]. It has been also adapted and validated in the rural Ethiopian setting among people with severe mental disorders [37]. The reliability and validity of the WHODAS-2.0 has been established with both local and cross-cultural validation studies [38–41].

The PHQ-9 was used to screen for depression. PHQ-9 is a widely used depression screening instrument in the PHC setting [42]. The items in the PHQ-9 ask about depressive symptoms present in the preceding 2 weeks. Each item in the PHQ-9 has four response categories which indicate the amount of time that the symptom was present, ranging from “not at all” to “nearly every day.” The PHQ-9 was validated in the PHC settings of the Butajira area (Meskan and Mareko districts) [43], which are neighboring districts to the Sodo district. It was also validated in a general hospital setting in Addis Ababa [44]. The general hospital study showed that the PHQ-9 has very good internal consistency, and test–retest reliability; and good sensitivity and specificity at a cut-off point of 10. The PHC study found very good internal consistency and good construct validity and convergent validity. In this study, the optimum cut-off point was determined to be five, with sensitivity 83.3% and specificity 74.7%. We used this cut-off point in the current study.

We assessed suicidality using the suicide items included in the Composite International Diagnostic Interview (CIDI). The questions within the CIDI relate to suicidal ideation, suicide plan and suicide attempt [45]. The CIDI is found to be feasible and acceptable and have good face validity and test–retest reliability [46]. It has been used in previous community based studies in the rural Ethiopian setting [47].

The Oslo social support scale (OSSS-3) was used to measure general social support. OSSS-3 is a three item scale which asks about number of close confidants, sense of concern or interest from other people and ease of getting practical help from neighbors [48]. The response categories are different for each of the three questions. OSSS-3 has been used in several studies and found to be feasible and have good predictive validity and convergent validity [49]. A total score, ranging from 3 to 14, can be created by adding up the raw score of each item. Although the scale was not validated in the Ethiopian setting, it has been used in previous community and facility-based studies and demonstrated good utility [30]. The List of Threatening Experiences (LTE), a brief questionnaire which is commonly used to assess stressful life events in epidemiological studies, was used to collect data on participants’ experience of stressful life events [50]. The LTE measures the occurrence of 12 prevalent major stressful events (e.g. death of a close relative or friend, loss of relationship, imprisonment and being the victim of theft) in the preceding 6 months, with dichotomous responses (yes/no). The LTE has been tested in over 15 languages and 20 different countries, and found to have good test–retest reliability and predictive validity [51]. It has been adapted and used for a rural Ethiopian setting [30].

The Alcohol Use Disorder Identification Test (AUDIT) was used to screen for problematic alcohol use. The AUDIT was developed by the WHO to assess alcohol consumption, drinking behaviors and alcohol related problems in the previous 12 months in people who are attending PHC facilities [52]. It has ten items, each rated in a four-point scale, giving a total score ranging from 0 to 40. The AUDIT has been validated across genders and in a wide range of cultural groups [53]. Local alcoholic beverages in the Ethiopian context have been converted into standard equivalent alcohol units [29]. A structured self-report demographic and socio-economic characteristics questionnaire was developed and administered to secure data related to the sex, age, urban–rural residence, ethnic group, religion, marital status, educational status, and socio-economic status of the participants.

For PHC diagnosis of depression, clinicians completed a clinical encounter form. This was a simple tool that allowed the clinician to record the presenting complaint, history of the presenting illness, any pertinent past history, findings of the physical examination, diagnosis, any investigations requested and treatment provided.

### Data management and analysis

Data were double entered in Epidata v3.0 and exported into STATA for windows (version 13) for analysis. Data cleaning was done using frequency distributions and logic checks, with reference to source documents as required. Frequencies and percentages were used to summarize variables which were categorical, whereas continuous variables were summarized using mean and standard deviation. Univariate and multivariable negative binomial regression models were fitted to assess the association of demographic, social, economic and clinical variables with functional impairment among people who were diagnosed to have depression. The same analyses were done to compare the functional impairment of people diagnosed to have depression (“PHC diagnosed cases”) to (i) those who had depressive symptoms, but who were not diagnosed to have depression (“non-diagnosed controls”), and (ii) community representative healthy controls, who had no depressive symptoms (“community controls”). Selected relevant demographic, social, economic and clinical characteristics were adjusted for in these analyses. Negative binomial regression was used because the distribution of WHODAS scores were skewed and only non-negative integer values are possible. Relative risk (RR), both crude and adjusted, with the corresponding 95% confidence interval, was used to estimate the strength of association between potential associated factors and the outcome variable in both the univariate and the multivariable models. All statistical tests were set at α = 0.05 for significance.

### Ethical considerations

Ethical approval was obtained from the Institutional Review Board of the College of Health Sciences, Addis Ababa University (Reference Number 084/11/Psy). Written informed consent was obtained from all the participants after the nature of the study and the information sought had been fully explained. Non-literate participants gave finger-prints to signify their willingness to participate. Participants with PHQ-9 scores higher than the cut-off point and those who reported suicidal ideation, plan or attempt were referred for mental health evaluation and possible treatment in their respective health centers.

## Results

### Characteristics of the participants

Study participants included 92 people diagnosed to have depression by PHC clinicians, 39 non-diagnosed facility-based controls and 197 community representative healthy controls. Details of the characteristics of the participants are presented in Table 1. The majority of the participants in PHC diagnosed cases (71.4%) and non-diagnosed controls (70.0%) were females; whereas the majority of the participants in community controls (78.7%) were males. The mean age in the PHC diagnosed cases, in the non-diagnosed controls and in the community controls was 38.1, 39.6 and 48.2 years, respectively. The majority of the PHC diagnosed cases (65.9%), the non-diagnosed controls (62.5%) and the community controls (82.7%) were married. Over three-quarters of the participants in PHC diagnosed cases (78.0%), non-diagnosed controls (80.0%) and community controls (79.8%) were rural residents. Only around a third of the participants in PHC diagnosed cases (35.4%) and in non-diagnosed controls (32.5%), and only 39.0% of the community controls attended formal education. Nearly 90% of the participants, in PHC diagnosed cases, non-diagnosed controls and community controls were from Gurage ethnic group and were Orthodox Christian by religion. There were no statistically significant differences between PHC diagnosed cases and non-diagnosed controls for any of the socio-demographic characteristics (P > 0.05).

**Table 1:** Socio-demographic and clinical characteristics of participants

Characteristics	PHC diagnosed cases (N = 92)	Non-diagnosed controls (N = 39)	Community controls (N = 197)
	N (%)	N (%)	N (%)
Socio-demographic characteristics			
Sex			
Female	66 (71.7)	27 (69.2)	42 (21.3)
Male	26 (28.3)	12 (30.8)	155 (78.7)
Age in years			
Mean (SD)	38.1 (14.1)	39.6 (15.8)	48.2 (13.4)
Marital status			
Never married	17 (18.5)	7 (17.9)	2 (1.0)
Currently married	60 (65.2)	25 (64.1)	162 (82.7)
Formerly married	15 (16.3)	7 (17.9)	32 (16.3)
Residence			
Rural	70 (76.1)	32 (82.1)	154 (79.8)
Urban	22 (23.9)	7 (17.9)	39 (20.2)
Educational status			
Cannot read and write	45 (48.9)	22 (56.4)	61 (31.0)
Able to read and write	14 (15.2)	5 (12.8)	59 (29.9)
Attend formal education	33 (35.9)	12 (30.8)	77 (39.1)
Number of years of education completed			
Mean (SD)	2.1 (3.3)	2.5 (3.7)	2.3 (3.8)
Ethnicity			
Gurage	82 (89.1)	37 (94.8)	183 (92.9)
Other	10 (10.9)	2 (5.1)	14 (7.1)
Religion			
Orthodox Christian	81 (88.0)	36 (92.3)	177 (89.8)
Other	11 (12.0)	3 (7.7)	20 (10.2)
Occupational status			
Farming	15 (16.3)	10 (25.6)	130 (66.0)
House wife	53 (57.6)	18 (46.2)	21 (10.7)
Other	24 (26.1)	11 (28.2)	46 (23.4)
Income level			
Lower	60 (65.2)	21 (53.8)	77 (39.1)
Middle or higher	32 (34.8)	18 (46.2)	120 (60.9)
Number of children			
No child	17 (18.5)	12 (30.8)	22 (11.2)
1–4 children	43 (46.7)	11 (28.2)	90 (45.7)
More than 4 children	32 (34.8)	16 (41.0)	85 (43.1)
Clinical characteristics			
Suicidal ideation			
Yes	54 (58.7)	8 (20.5)	–
No	38 (41.3)	31 (79.5)	
Suicidal plan			
Yes	29 (31.5)	8 (20.5)	–
No	63 (68.5)	31 (79.5)	
Suicidal attempt			
Yes	17 (18.5)	7 (17.9)	–
No	75 (81.5)	32 (82.1)	
Depressive symptom score			
Mean (SD)	10.46 (4.56)	8.93 (3.44)	1.64 (1.46)
Functional impairment score			
Mean (SD)	26.81 (9.13)	25.15 (8.51)	14.56 (4.14)
Alcohol use disorder score			
Mean (SD)	9.03 (8.14)	6.40 (6.63)	–
Social support total score			
Mean (SD)	9.57 (2.88)	10.73 (1.80)	–
Threatening life events score			
Mean (SD)	1.90 (2.06)	2.10 (1.79)	–

*PHC* primary healthcare, *SD* standard deviation, *PHQ* Patient Health Questionnaire, *WHODAS* WHO Disability Assessment Schedule

The mean PHQ score was significantly higher in PHC diagnosed cases (mean = 10.46 and standard deviation [SD] = 4.56) compared to non-diagnosed controls (mean = 8.93 and SD = 3.44); P < 0.05. Suicidal ideation but not plans or attempts was significantly higher in the PHC diagnosed cases compared to non-diagnosed controls. Around 21% of those who had suicidal ideation and plan and 18% of those who had suicidal attempt were not diagnosed to have depression by PHC clinicians. The mean AUDIT total score was also significantly higher in the PHC diagnosed cases (9.03; SD = 8.14) compared to non-diagnosed controls (6.40; SD = 6.63); P < 0.05. The mean social support score, however, was significantly lower in the PHC diagnosed cases (9.57; SD = 2.88) compared to non-diagnosed controls (10.73; SD = 1.80); P < 0.05. There was no statistically significant difference in mean WHODAS-2.0 score or number of stressful life events in the PHC diagnosed cases compared to the non-diagnosed controls.

### Association of depression diagnosis with WHODAS-2.0 scores among PHC diagnosed and non-diagnosed cases

PHC depression diagnosis, the dependent variable in this regression model, in people with a positive PHQ screen was not significantly associated with functional impairment, either in the univariate (RR = 1.07; 95% CI 0.94, 1.21) or multivariable models (RR = 0.96; 95% CI 0.87, 1.06). See Table 2.

**Table 2:** The association of depression diagnosis with WHODAS-2.0 scores among PHC diagnosed and non-diagnosed cases (n = 131)

Variables	CRR (95% CI)	ARR (95% CI)
Sex		
Male	1.0	1.0
Female	0.91 [0.80, 1.03]	1.02 [0.90, 1.15]
Age in years	1.00 (0.99, 1.01]	1.00 [0.99, 1.01)
Marital status		
Never married	1.0	1.0
Currently married	0.90 [0.77, 1.04]	1.07 [0.90, 1.29]
Formerly married	0.99 [0.82, 1.21]	1.06 [0.87, 1.30]
Residence		
Urban	1.0	1.0
Rural	*0.82 [0.72, 0.94]*	0.90 [0.80, 1.02]
Number of years of education completed	0.99 [0.98, 1.01]	*0.98 [0.96, 0.99]*
Number of children participants have		
No child	1.0	1.0
1–4 children	0.93 [0.80, 1.08]	0.83 [0.68, 1.01]
More than 4 children	0.95 [0.81, 1.11]	0.88 [0.72, 1.08]
Income level		
Lower	1.0	1.0
Middle or higher	0.90 [0.80, 1.02]	0.97 [0.88, 1.06]
Economic deprivation	*1.07 [1.01, 1.13]*	1.04 [0.99, 1.09]
Alcohol use disorder symptom score	1.00 [0.99, 1.01]	1.00 [0.99, 1.01]
Stressful life events	*1.04 [1.01, 1.07]*	1.01 [0.99, 1.03]
Social support	*0.95 [0.93, 0.96]*	*0.96 [0.94, 0.97]*
Depressive symptom score	*1.04 [1.03, 1.06]*	*1.04 [1.03, 1.05]*
PHC depression diagnosis		
Controls	1.0	1.0
PHC diagnosed cases	1.07 [0.94, 1.21]	0.96 [0.87, 1.06]
Suicidal ideation		
No	1.0	1.0
Yes	1.11 [0.99, 1.24]	1.01 [0.91, 1.13]
Suicidal plan		
No	1.0	1.0
Yes	1.04 [0.92, 1.18]	0.97 [0.82, 1.14]
Suicidal attempt		
No	1.0	1.0
Yes	1.02 [0.87, 1.17]	0.94 [0.79, 1.12]

Values in italics indicate statistical significance

*CRR* crude relative risk, *ARR* adjusted relative risk, *WHODAS* WHO Disability Assessment Schedule, AUDIT alcohol use disorder identification test, *LTE* list of threatening experiences, *OSSS* Oslo Social Support Scale, *PHQ* Patient Health Questionnaire, *PHC* Primary Healthcare

In the multivariable model, higher depression symptom scores were associated with greater functional impairment (RR = 1.04; CI 1.03, 1.05); whereas better social support (RR = 0.96; CI 0.94, 0.97) and higher number of years of education completed (RR = 0.98; CI 0.96, 0.99) were significantly associated with lower functional impairment. In the univariate models, economic deprivation (RR = 1.07; CI 1.01, 1.13) and stressful life events (RR = 1.04; CI 1.01, 1.07) were significantly associated with greater functional impairment; whereas rural residence (RR = 0.82; CI 0.72, 0.94) was significantly associated with lower functional impairment. Nevertheless, these univariate associations were not maintained in the multivariable model.

### Factors associated with WHODAS-2.0 scores among PHC diagnosed cases only

In the univariate models, economic deprivation or poverty (RR = 1.08; 95% CI 1.00, 1.15) and experiencing threatening life events (RR = 1.05; 95% CI 1.01; 1.08) were associated with greater functional impairment; whereas rural residence (RR = 0.78; 955 CI 0.67, 0.92) was associated with lower functional impairment. However, these associations became non-significant in the multivariable model. In the multivariable model, better social support (RR = 0.96; 95% CI 0.95, 0.98) was significantly associated with lower functional impairment. Higher depression symptom severity was associated with greater functional impairment (RR = 1.04; 95% CI 1.02, 1.05). See Table 3.

**Table 3:** The association of demographic, social, economic and clinical factors with WHODAS-2.0 scores among primary healthcare diagnosed cases only (n = 92)

Variables	CRR (95% CI)	ARR (95% CI)
Sex		
Male	1.0	1.0
Female	0.88 [0.76, 1.03]	0.98 [0.83, 1.15]
Age in years	1.00 (0.99, 1.01]	1.00 [0.99, 1.01)
Marital status		
Never married	1.0	1.0
Currently married	0.93 [0.77, 1.11]	1.19 [0.91, 1.55]
Formerly married	1.08 [0.86, 1.37]	1.20 [0.92, 1.56]
Residence		
Urban	1.0	1.0
Rural	*0.78 [0.67, 0.92]*	0.88 [0.75, 1.02]
Number of years of education completed	1.01 [0.98, 1.03]	0.99 [0.96, 1.01]
Number of children participants have		
No child	1.0	1.0
1–4 children	0.92 [0.76, 1.12]	0.85 [0.64, 1.14]
More than 4 children	0.93 [0.76, 1.14]	0.89 [0.66, 1.19]
Income level		
Lower	1.0	1.0
Middle or higher	0.88 [0.76, 1.02]	0.95 [0.84, 1.08]
Economic deprivation	*1.08 [1.00, 1.15]*	1.05 [0.98, 1.12]
Alcohol use disorder symptom score	1.00 [0.99, 1.01]	1.00 [0.99, 1.01]
Stressful life events	*1.05 [1.01, 1.08]*	1.01 [0.98, 1.04]
Social support	*0.95 [0.93, 0.97]*	*0.96 [0.95, 0.98]*
Depressive symptom score	*1.04 [1.03, 1.05]*	*1.04 [1.02, 1.05]*

Values in italics indicate statistical significance

*CRR* crude relative risk, ARR adjusted relative risk, *WHODAS* WHO Disability Assessment Schedule, *AUDIT* Alcohol Use Disorder Identification Test*, LTE* List of Threatening Experiences, *OSSS* Oslo Social Support Scale*, PHQ* Patient Health Questionnaire

### Functional impairment in PHC diagnosed cases vs. community controls

Results of the univariate and multivariable models of WHODAS-2.0 scores among PHC diagnosed cases and community controls are presented in Table 4. Having a PHC clinician diagnosis of depression was significantly associated with greater functional impairment, both before and after adjustment: RR = 1.91; 95% CI 1.74, 2.09.

**Table 4:** Association of depression status with WHODAS-2.0 scores among primary healthcare diagnosed cases and community controls (n = 289)

Variables	CRR (95% CI)	ARR (95% CI)
Depression status		
Healthy controls	1.0	1.0
Cases	*1.84 [1.71, 1.98]*	*1.91 [1.74, 2.09]*
Sex		
Male	1.0	1.0
Female	*1.36 [1.24, 1.49]*	0.96 [0.87, 1.06]
Age	1.00 (0.99, 1.00]	*1.00 [1.00, 1.01]*
Marital status		
Never married	1.0	1.0
Currently married	*0.66 [0.55, 0.79]*	0.96 [0.81, 1.13]
Formerly married	*0.81 [0.65, 0.99]*	1.08 [0.89, 1.31]
Residence		
Urban	1.0	1.0
Rural	*0.85 [0.76, 0.96]*	*0.84 [0.77, 0.92]*
Number of years of education completed	0.99 [0.98, 1.01]	0.99 [0.98, 1.00]
Number of children participants have		
No child	1.0	1.0
1–4 children	0.91 [0.79, 1.06]	0.93 [0.81, 1.06]
More than 4 children	0.91 [0.78, 1.05]	0.97 [0.85, 1.11]
Income level		
Lower	1.0	1.0
Middle or higher	*0.79 [0.72, 0.87]*	0.95 [0.89, 1.03]

Values in italics indicate statistical significance

*CRR* crude relative risk, *ARR* adjusted relative risk, *WHODAS* WHO Disability Assessment Schedule

In the univariate models, being currently married (RR = 0.66; 95% CI 0.55, 0.79), being formerly married (RR = 0.81; 95% CI 0.65, 0.99) and perceiving one’s own economic status as middle or higher (RR = 0.79; 95% CI 0.72, 0.87) were significantly associated with lower functional impairment. Female gender (RR = 1.36; 95% CI 1.24, 1.49) was significantly associated with greater functional impairment. In the multivariable model, however, older age (RR = 1.00; 95% CI 1.00, 1.01) was associated with greater functional impairment, whereas rural residence (RR = 0.84; 95% CI 0.77, 0.92) was significantly associated with lower functional impairment.

## Discussion

In this comparative cross-sectional study in a rural low income setting where a task-sharing model of mental health care had been newly implemented, PHC clinician diagnosis of depression, in people with a positive PHQ screen, was associated with depressive symptom severity, suicidal ideation and co-morbid alcohol use disorder, but not with degree of functional impairment. In the people with a PHC diagnosis of depression, functional impairment was associated with worse depressive symptom severity and poorer social support. Compared to community controls, people with a PHC diagnosis of depression had significantly greater functional impairment.

Our study findings provide some evidence of the validity of depression diagnosis by PHC clinicians in this setting and support the approach to expanding access to mental health care. In the mhGAP training for primary health care workers, the importance of functional impairment for diagnosis of depression is emphasized, particularly in relation to initiation of pharmaceutical treatment [1]. A major potential concern about a task-sharing approach to care for people with depression is that non-specialist workers might medicalise symptoms of transient distress or social adversity by attributing to depression [54]. That does not seem to have been the case in our study; indeed, some people with significant morbidity (e.g. suicidal ideation) did not receive a diagnosis of depression. This requires further exploration to see if PHC clinicians adequately assessed suicidality or whether this reflects false positives for suicidality using the fully structured instruments or PHC clinician recognition of a sub-group of people with suicidal ideation who do not have underlying depression.

In the people with a PHC diagnosis of depression, the significant association between severity of depressive symptoms and greater functional impairment is in keeping with our hypothesis and consistent with the literature. A recent systematic review of the available evidence [15] showed that lower severity of depressive symptoms, shorter duration of the current depressive episode and shorter duration of untreated depression are associated with better functional outcome. Analysis of data of a large sample of adult outpatients with major depressive disorder at entry and exit points of antidepressant treatment showed that only 7% of patients with depression symptoms reported within-normal functioning at baseline [55]. In a population-based study of psychiatric morbidity and functional impairment among disaster victims in Norway, depressive symptom severity was associated with both self-reported and clinician assessed functional impairment [14].

The association of higher perceived social support with lower functional impairment in the current study is also in keeping with expectations. Research on the association between social support and functional impairment among people with depressive symptoms is generally scarce particularly in the low income setting. One of the explanations for this association may be that the emotional and instrumental support, encouragement and push from significant others would help people with depression to gain energy and motivation for accomplishing their day to day activities. The other possible explanation could be that higher scores in the social support measure may increase scores in the social participation domain of the disability measure. Studies from both high income countries [56] and LMICs [30] showed that lower perceived social support is significantly associated with higher depressive symptom scores, which may have the implication that social support improves functional capacity through reducing depressive symptoms. In a longitudinal study designed to determine the mediating role of proactive coping in the relationship between social support and functional outcome in people with severe mental illnesses, Davis and Brekke [57] concluded that social support facilitates proactive coping processes to enhance role functioning. In our previous qualitative study conducted in a similar setting with the current study, service user, caregiver and health care worker participants reported that supporting and encouraging people with severe mental illnesses improves their ability to accomplish daily activities, work and social responsibilities [58].

In the larger sample which combined PHC diagnosed cases and community controls, we found that older age and urban residence were independently associated with greater functional impairment. Overall, these findings are consistent with previous studies conducted in people with major depressive disorder [20] and schizophrenia [59]. Functional impairment and disability would increase as one gets older due to reduced social relationship, loss of natural supports, deterioration of physical health and reduced independent living skill. The finding that people with depression from rural areas have lower functional impairment compared to urban residents is consistent with findings from previous studies [60]. This may be because family support, arranged marriage and availability of less stressful work settings are more prevalent in rural areas than in urban settings. Arranged marriage is common in rural areas more than in urban settings of Ethiopia, and this is likely to increase the social functioning of people with depression who are from rural areas. In addition, participating in social activities and working in the farm are crucial for survival of the person with mental illness living in a rural area and his/her family members.

Univariate significant association was found between indicators of economic deprivation and functional impairment; however this association was not maintained in the multivariable model. One of the reasons for this may be the small sample size in the analysis using the sample of PHC diagnosed cases. In the analysis with the larger sample, middle or higher perceived wealth relative to neighbors was significantly associated with lower functional impairment in the univariate model. Overall, the study showed that economic deprivation or poverty is an important factor contributing to disability among people diagnosed with depression and community healthy controls. Previous studies found that lower socio-economic position and economic deprivation were associated with lower social participation and interpersonal relationship [61]. Studies also indicate that higher socio-economic status is associated with functional recovery in people with severe mental illnesses [62].

This study indicates that people with depression have a significant burden in this rural low income African setting. Assessing and treating patients attending PHC for depressive symptoms may help to reduce the burden of depression. This in turn requires integrating mental health services in the PHC setting. There are previous studies in LMICs which indicated that depression can be effectively treated in the PHC setting by general health care professionals [63] and that integrating mental health services in the PHC level is feasible and acceptable [64]. The current study also indicates that social support would help people with depression to improve their functional recovery. Involving caregivers and family members in the treatment of depression, therefore, may play an important role to facilitate functional improvement. It would also be important for mental health care providers to give priority for older people and for the poorest part of the population since these groups are vulnerable for both depression and functional impairment.

## Strengths and limitations

One of the strengths of this study is that we included two types of controls to compare the functional impairment of PHC diagnosed depression cases (facility-based non-diagnosed controls but screened to have depression and community representative healthy controls). The other major strength of the study is that the primary exposure variable and the outcome variable were measured using locally validated instruments (PHQ-9 and WHODAS-2.0) which have very good psychometric properties. However, the findings should be interpreted acknowledging certain important limitations. The main sample of this study (PHC diagnosed cases) was recruited from attendees in primary care and may not be representative of the general population. The study is cross-sectional, and therefore the direction of association cannot be determined. The other important limitation of our study is the small sample size that we used to determine the association of demographic, social, economic and clinical characteristics with functional impairment in people who have diagnosed depression.

## Conclusions

Our findings support the validity of depression diagnosis made by PHC clinicians after brief training in a rural African setting. Social support may be an important target for intervention to improve functional impairment in people with depressive symptoms. There is a need for follow-up studies to compare the outcomes of people with high depressive symptoms who are diagnosed vs. those who do not get a diagnosis of depression. In PRIME, we will evaluate the impact of task-shared care on clinical, social and functional outcomes in people diagnosed by PHC clinicians as having depression.

## Data Availability

This study is part of a larger collaborative study, the PRIME project. Data used for this analysis will become available through the PRIME project.

## Abbreviations

AUDIT: The Alcohol Use Disorder Identification Test
CI: confidence interval
CIDI: Composite International Diagnostic Interview
LMICs: low and middle income countries
LTE: The List of Threatening Experiences
PHC: primary healthcare
PHQ-9: 9-item Patient Health Questionnaire
PRIME: Programme for Improving Mental Health Care
OSSS-3: The Oslo social support scale
RR: relative risk
SMD: severe mental disorder
SNNPR: Southern Nations, Nationalities and People’s Region
WHO: World Health Organization
WHODAS-2.0: WHO Disability Assessment Schedule
